# Slicing through the challenge of maintaining *Pneumocystis* in the laboratory

**DOI:** 10.1128/mbio.03277-23

**Published:** 2024-02-12

**Authors:** Olga A. Nev, Lucian Duvenage, Alistair J. P. Brown, Rachael Dangarembizi, Jennifer Claire Hoving

**Affiliations:** 1 Biosciences and Living Systems Institute, University of Exeter, Exeter, United Kingdom; 2 Medical Research Council Centre for Medical Mycology, University of Exeter, Exeter, United Kingdom; 3 CMM AFRICA Medical Mycology Research Unit, Institute of Infectious Diseases and Molecular Medicine, University of Cape Town, Cape Town, South Africa; 4 Department of Pathology, Division of Immunology, Faculty of Health Sciences, University of Cape Town, Cape Town, South Africa; 5 Department of Human Biology, Division of Physiological Sciences, Faculty of Health Sciences, University of Cape Town, Cape Town, South Africa; 6 Neuroscience Institute, Faculty of Health Sciences, University of Cape Town, Groote Schuur Hospital, Cape Town, South Africa; Duke University, Durham, North Carolina, USA

**Keywords:** *Pneumocystis*, fungal pathogenicity, medical mycology, precision-cut lung slices

## Abstract

*Pneumocystis jirovecii* is a major fungal pathogen of humans that causes life-threatening lung infections in immunocompromised individuals. Despite its huge global impact upon human health, our understanding of the pathobiology of this deadly fungus remains extremely limited, largely because it is not yet possible to cultivate *Pneumocystis in vitro,* independently of the host. However, a recent paper by Munyonho et al. offers a major step forward (F. T. Munyonho, R. D. Clark, D. Lin, M. S. Khatun, et al., 2023, mBio 15:e01464-23, https://doi.org/10.1128/mbio.01464-23). They show that it is possible to maintain both the trophozoite and cyst forms of the mouse pathogen, *Pneumocystis murina,* in precision-cut lung slices for several weeks. Furthermore, they demonstrate that this offers the exciting opportunity to examine potential virulence factors such as possible biofilm formation as well as antifungal drug responses in the lung.

## COMMENTARY

*Pneumocystis jirovecii* is a deadly pathogen of humans that causes life-threatening pneumonia in immunocompromised individuals. This fungus is found in the lungs of infants (1, 2) and is known to cause fatal pneumonia in patients with compromised immune responses, including transplant recipients (3, 4). It continues to be one of the most prevalent and severe infections among individuals with HIV/AIDS (5), representing a significant health challenge, particularly within the developing nations of sub-Saharan Africa. In addition to HIV/AIDS, a variety of other factors that promote immunosuppression have emerged as significant risk factors for *Pneumocystis* pneumonia (PCP) (6, 7). Moreover, PCP is becoming increasingly problematic for non-HIV-infected individuals in developed countries, including Sweden (8), the United Kingdom (9), and the United States (10). Despite the major impact of *P. jirovecii* on global health, our understanding of the biology of this fungus remains alarmingly limited. This is largely because significant challenges, primarily the lack of robust *in vitro* and *ex vivo* models, have severely restricted the experimental dissection of the pathogenesis of *Pneumocystis* species. Indeed, the absence of robust cultivation techniques is widely acknowledged to be the major obstacle in *Pneumocystis* research. Thus far, attempts to bypass likely auxotrophic requirements (predicted on the basis of genome sequence analyses) through supplementation of culture media have not proven sufficient to facilitate growth *in vitro* (11).

Therefore, the paper entitled “Precision cut lung slices as an *ex vivo* model to study *Pneumocystis murina* survival and antimicrobial susceptibility” represents an exciting step forward (12). In this paper, Munyonho et al. describe the development of an innovative *ex vivo* model, based on the use of precision-cut lung slices (PCLS) from mice, to study the survival of the mouse pathogen *Pneumocystis murina*. Using a combination of cellular, molecular, and histochemical approaches, they firstly confirmed the viability of the lung slices over a 15-day period. Their thin lung sections maintained the structural and functional characteristics of lung tissue, thereby providing a physiologically relevant environment for the pathogen by replicating the alveolar space of the host lung where *Pneumocystis* predominantly resides (13). Then, using a combination of reverse transcriptase-quantitative polymerase chain reaction and immunohistochemistry, Munyonho et al. demonstrated that, over this period, both the cyst and trophozoite forms of the fungus retained their viability in these lung slices but not in Dulbecco’s modified Eagle’s medium alone. This was the case for lung slices from immunocompetent (C57BL/6) as well as immunodeficient (*Rag2*^−/^*^−^ Il2rγ^−^*^/^*^−^*) mice. Having established this ground-breaking *ex vivo* PCLS system, the authors then highlight its potential applications by examining the susceptibility of *P. murina* to commonly used antifungal treatments such as trimethoprim-sulfamethoxazole and echinocandins (14, 15).

This important paper represents a significant breakthrough for the *Pneumocystis* research community and medical mycologists at large. From an academic perspective, the observation of Munyonho et al. that *P. murina* forms fungal aggregates, potentially biofilms, in the tissue slices is intriguing. This observation, which is consistent with previous reports (16), paves the way toward the experimental dissection of this phenomenon as well as the mechanisms by which *Pneumocystis* species adhere to and scavenge from host tissue. Some limitations, such as the lack of a systemic immune response in the tissue slices, will influence their utility in the dissection of antifungal immune responses. Nevertheless, the native lung environment and the heterogeneity of resident cells are preserved in PCLS. For example, alveolar macrophages and alveolar dendritic cells are critical for the immune response against *Pneumocystis* (17, 18), and imaging of these cells in lung slices reveals similar antigen uptake and presentation when compared with *in vivo* imaging techniques such as intravital stabilized lung imaging (19). Looking forward, the use of PCLS in conjunction with advanced microfluidics and live imaging is likely to facilitate studies of the dynamics of immune recognition of *Pneumocystis* while reducing animal usage. Transcriptomic analyses of infected PCLS are likely to provide complementary information about *Pneumocystis* pathobiology and immune activation (20). The growth of *Pneumocystis* on PCLS might even empower forward genetics to yield mutants capable of independent growth on appropriately supplemented media *in vitro*.

Precision-cut tissue slices can also be prepared from organs such as the brain, liver, and pancreas, as well as from different species including rodents, monkeys, and humans. Indeed, in several recent studies, PCLS from a variety of species, including human, have been used to elucidate the adherence of other microbial pathogens and local cytokine responses against these pathogens (21–24). Therefore, from a clinical perspective, this new paper adds to the recent successful application of *ex vivo* PCLS systems for modeling lung disorders (25, 26) and for studies of asthma (27), chronic obstructive pulmonary disease (COPD) (28), and idiopathic pulmonary fibrosis (29). Organotypic brain slice cultures have also been used successfully to study neuroimmune responses to brain infections with *Cryptococcus neoformans* (30), a fungal pathogen that usually infects the lung initially but later disseminates to cause injury to the brain. *Pneumocystis* infections of the central nervous system have been reported in patients with advanced HIV disease (31, 32), but these remain understudied. Therefore, the development of organ-specific approaches for *Pneumocystis* also provides a unique opportunity to study fungus-host interactions in extra-pulmonary infections. Also, the availability of surgically resected lung and brain tissue offers exciting translational opportunities to use precision-cut tissue slices to study human-specific host-pathogen interactions, as well as to streamline the evaluation of new antifungal agents, thereby accelerating the pace of therapeutic advancements.

In summary, the study by Munyonho et al. represents an exciting step forward in *Pneumocystis* research. The development of a viable *ex vivo* model using PCLS will not only enrich our understanding of *Pneumocystis* biology but will also potentially shape how we approach PCP treatment and prevention.

## References

[1] Beard CB, Fox MR, Lawrence GG, Guarner J, Hanzlick RL, Huang L, del Rio C, Rimland D, Duchin JS, Colley DG. 2005. Genetic differences in Pneumocystis isolates recovered from immunocompetent infants and from adults with AIDS: epidemiological implications. J Infect Dis 192:1815–1818. doi:10.1086/497381 16235182

[2] Vargas SL, Hughes WT, Santolaya ME, Ulloa AV, Ponce CA, Cabrera CE, Cumsille F, Gigliotti F. 2001. Search for primary infection by Pneumocystis carinii in a cohort of normal, healthy infants. Clin Infect Dis 32:855–861. doi:10.1086/319340 11247708

[3] de Boer MGJ, de Fijter JW, Kroon FP. 2011. Outbreaks and clustering of Pneumocystis pneumonia in kidney transplant recipients: a systematic review. Med Mycol 49:673–680. doi:10.3109/13693786.2011.571294 21453224

[4] Yiannakis EP, Boswell TC. 2016. Systematic review of outbreaks of Pneumocystis jirovecii pneumonia: evidence that P. jirovecii is a transmissible organism and the implications for healthcare infection control. J Hosp Infect 93:1–8. doi:10.1016/j.jhin.2016.01.018 26996089

[5] Buchacz K, Lau B, Jing Y, Bosch R, Abraham AG, Gill MJ, Silverberg MJ, Goedert JJ, Sterling TR, Althoff KN, Martin JN, Burkholder G, Gandhi N, Samji H, Patel P, Rachlis A, Thorne JE, Napravnik S, Henry K, Mayor A, Gebo K, Gange SJ, Moore RD, Brooks JT. 2016. Incidence of AIDS-defining opportunistic infections in a multicohort analysis of HIV-infected persons in the United States and Canada, 2000–2010. J Infect Dis 214:862–872. doi:10.1093/infdis/jiw085 27559122 PMC4996145

[6] Yale SH, Limper AH. 1996. Pneumocystis carinii pneumonia in patients without acquired immunodeficiency syndrome: associated illness and prior corticosteroid therapy. Mayo Clin Proc 71:5–13. doi:10.4065/71.1.5 8538233

[7] Enomoto T, Azuma A, Kohno A, Kaneko K, Saito H, Kametaka M, Usuki J, Gemma A, Kudoh S, Nakamura S. 2010. Differences in the clinical characteristics of Pneumocystis jirovecii pneumonia in immunocompromized patients with and without HIV infection. Respirology 15:126–131. doi:10.1111/j.1440-1843.2009.01660.x 19947989

[8] Mikaelsson L, Jacobsson G, Andersson R. 2006. Pneumocystis pneumonia—a retrospective study 1991–2001 in Gothenburg, Sweden. J Infect 53:260–265. doi:10.1016/j.jinf.2005.06.014 16403575

[9] Maini R, Katherine LH, Elizabeth AS, Theresa L, Gordon N, Valerie D, Nick P. 2000. Increasing Pneumocystis pneumonia, England, UK, 2000–2010. Emerging Infect Dis 19:386–392. doi:10.3201/eid1903.121151 PMC3647665 23622345

[10] Inagaki K, Blackshear C, Hobbs CV. 2019. Pneumocystis infection in children: national trends and characteristics in the United States, 1997–2012. Pediatr Infect Dis J 38:241–247. doi:10.1097/INF.0000000000002119 29794652

[11] Cushion MT, Tisdale-Macioce N, Sayson SG, Porollo A. 2021. The persistent challenge of Pneumocystis growth outside the mammalian lung: past and future approaches. Front Microbiol 12:681474. doi:10.3389/fmicb.2021.681474 34093506 PMC8174303

[12] Munyonho FT, Clark RDE, Lin D, Khatun MS, Pungan D, Dai G, Kolls JK. 2023. Precision-cut lung slices as an ex vivo model to study Pneumocystis murina survival and antimicrobial susceptibility. mBio 15:e0146423. doi:10.1128/mbio.01464-23 38117035 PMC10790776

[13] Skalski JH, Kottom TJ, Limper AH. 2015. Pathobiology of Pneumocystis pneumonia: life cycle, cell wall and cell signal transduction. FEMS Yeast Res 15:fov046. doi:10.1093/femsyr/fov046 26071598

[14] Carmona EM, Limper AH. 2011. Update on the diagnosis and treatment of Pneumocystis pneumonia. Ther Adv Respir Dis 5:41–59. doi:10.1177/1753465810380102 20736243 PMC6886706

[15] Safrin S, Finkelstein DM, Feinberg J, Frame P, Simpson G, Wu A, Cheung T, Soeiro R, Hojczyk P, Black JR. 1996. Comparison of three regimens for treatment of mild to moderate Pneumocystis carinii pneumonia in patients with AIDS: a double-blind, randomized trial of oral trimethoprim-sulfamethoxazole, dapsone-trimethoprim, and clindamycin-primaquine. Ann Intern Med 124:792–802. doi:10.7326/0003-4819-124-9-199605010-00003 8610948

[16] Cushion MT, Collins MS, Linke MJ. 2009. Biofilm formation by Pneumocystis spp. Eukaryot Cell 8:197–206. doi:10.1128/EC.00202-08 18820078 PMC2643605

[17] Carmona EM, Kottom TJ, Hebrink DM, Moua T, Singh R-D, Pagano RE, Limper AH. 2012. Glycosphingolipids mediate Pneumocystis cell wall β-glucan activation of the IL-23/IL-17 axis in human dendritic cells. Am J Respir Cell Mol Biol 47:50–59. doi:10.1165/rcmb.2011-0159OC 22343219 PMC3402796

[18] Limper AH, Hoyte JS, Standing JE. 1997. The role of alveolar macrophages in Pneumocystis carinii degradation and clearance from the lung. J Clin Invest 99:2110–2117. doi:10.1172/JCI119384 9151783 PMC508041

[19] Thornton EE, Looney MR, Bose O, Sen D, Sheppard D, Locksley R, Huang X, Krummel MF. 2012. Spatiotemporally separated antigen uptake by alveolar dendritic cells and airway presentation to T cells in the lung. J Exp Med 209:1183–1199. doi:10.1084/jem.20112667 22585735 PMC3371730

[20] Niehof M, Hildebrandt T, Danov O, Arndt K, Koschmann J, Dahlmann F, Hansen T, Sewald K. 2017. RNA isolation from precision-cut lung slices (PCLS) from different species. BMC Res Notes 10:121. doi:10.1186/s13104-017-2447-6 28274266 PMC5343379

[21] Weldearegay YB, Müller S, Hänske J, Schulze A, Kostka A, Rüger N, Hewicker-Trautwein M, Brehm R, Valentin-Weigand P, Kammerer R, Jores J, Meens J. 2019. Host-pathogen interactions of Mycoplasma mycoides in caprine and bovine precision-cut lung slices (PCLS) models. Pathogens 8:82. doi:10.3390/pathogens8020082 31226867 PMC6631151

[22] Kolbe U, Yi B, Poth T, Saunders A, Boutin S, Dalpke AH. 2020. Early cytokine induction upon Pseudomonas aeruginosa infection in murine precision cut lung slices depends on sensing of bacterial viability. Front Immunol 11:598636. doi:10.3389/fimmu.2020.598636 33250899 PMC7673395

[23] Bryson KJ, Garrido D, Esposito M, McLachlan G, Digard P, Schouler C, Guabiraba R, Trapp S, Vervelde L. 2020. Precision cut lung slices: a novel versatile tool to examine host-pathogen interaction in the chicken lung. Vet Res 51:2. doi:10.1186/s13567-019-0733-0 31924278 PMC6954617

[24] Brann KR, Fullerton MS, Onyilagha FI, Prince AA, Kurten RC, Rom JS, Blevins JS, Smeltzer MS, Voth DE. 2019. Infection of primary human alveolar macrophages alters Staphylococcus aureus toxin production and activity. Infect Immun 87:e00167-19. doi:10.1128/IAI.00167-19 31010814 PMC6589068

[25] Viana F, O’Kane CM, Schroeder GN. 2022. Precision-cut lung slices: a powerful ex vivo model to investigate respiratory infectious diseases. Mol Microbiol 117:578–588. doi:10.1111/mmi.14817 34570407 PMC9298270

[26] Liu G, Betts C, Cunoosamy DM, Åberg PM, Hornberg JJ, Sivars KB, Cohen TS. 2019. Use of precision cut lung slices as a translational model for the study of lung biology. Respir Res 20:162. doi:10.1186/s12931-019-1131-x 31324219 PMC6642541

[27] Cooper PR, Zhang J, Damera G, Hoshi T, Zopf DA, Panettieri RA. 2011. C-027 inhibits IgE-mediated passive sensitization bronchoconstriction and acts as a histamine and serotonin antagonist in human airways. Allergy Asthma Proc 32:359–365. doi:10.2500/aap.2011.32.3460 22195688 PMC3968313

[28] Van Dijk EM, Culha S, Menzen MH, Bidan CM, Gosens R. 2016. Elastase-induced parenchymal disruption and airway hyper responsiveness in mouse precision cut lung slices: toward an ex vivo COPD model. Front Physiol 7:657. doi:10.3389/fphys.2016.00657 28101062 PMC5209351

[29] Alsafadi HN, Staab-Weijnitz CA, Lehmann M, Lindner M, Peschel B, Königshoff M, Wagner DE. 2017. An ex vivo model to induce early fibrosis-like changes in human precision-cut lung slices. Am J Physiol Lung Cell Mol Physiol 312:L896–L902. doi:10.1152/ajplung.00084.2017 28314802

[30] Awala AN, Kauchali M, de Lange A, Higgitt ER, Mbangiwa T, Raimondo JV, Dangarembizi R. 2023. Mouse organotypic brain slice cultures: a novel model for studying neuroimmune responses to cryptococcal brain infections. Methods Mol Biol 2667:31–45. doi:10.1007/978-1-0716-3199-7_3 37145274

[31] Nasnas R, Matta MY, El Helou G, Rizk N, Sarkis DK, Moussa R. 2012. Brain abscesses in HIV-positive patients due to Pneumocystis jiroveci. J Int Assoc Physicians AIDS Care 11:169–171. doi:10.1177/1545109712439625 22453876

[32] Vidal F, Mirón M, Sirvent JJ, Richart C. 2000. Central nervous system pneumocystosis in AIDS: antemortem diagnosis and successful treatment. Clin Infect Dis 30:397–398. doi:10.1086/313679 10671351

